# Association of Genetic Variances in *ADRB1* and *PPARGC1a* with Two-Kilometre Running Time-Trial Performance in Australian Football League Players: A Preliminary Study

**DOI:** 10.3390/sports9020022

**Published:** 2021-01-29

**Authors:** Ysabel Jacob, Ryan S. Anderton, Jodie L. Cochrane Wilkie, Brent Rogalski, Simon M. Laws, Anthony Jones, Tania Spiteri, Nicolas H. Hart

**Affiliations:** 1 School of Medical and Health Sciences, Edith Cowan University, Perth 6027, Australia; y.jacob@ecu.edu.au (Y.J.); j.wilkie@ecu.edu.au (J.L.C.W.); s.laws@ecu.edu.au (S.M.L.); t.spiteri@ecu.edu.au (T.S.); 2 Institute for Health Research, University of Notre Dame Australia, Perth 6160, Australia; 3 School of Health Science, University of Notre Dame Australia, Perth 6160, Australia; 4 Perron Institute for Neurological and Translational Science, Perth 6009, Australia; 5 Centre for Exercise and Sport Science Research, Edith Cowan University, Perth 6027, Australia; 6 West Coast Eagles Football Club, Perth 6100, Australia; brentr@wce.com.au (B.R.); anthonyj@wce.com.au (A.J.); 7 Collaborative Genomics Group, School of Medical and Health Sciences, Edith Cowan University, Perth 6027, Australia; 8 Faculty of Health Sciences, School of Pharmacy and Biomedical Sciences, Curtin Health Innovation Research Institute, Curtin University, Perth 6102, Australia; 9 Exercise Medicine Research Institute, Edith Cowan University, Perth 6027, Australia; 10 Faculty of Health, Queensland University of Technology, Brisbane 4059, Australia

**Keywords:** *ADRB1*, *PPARGC1a*, genes, sport, performance, Australian Football, endurance

## Abstract

Genetic variants in the *angiotensin-converting enzyme* (*ACE*) (rs4343), *alpha-actinin-3* (*ACTN3*) (rs1815739), *adrenoceptor-beta-1* (*ADRB1*) (rs1801253), and *peroxisome proliferator-activated receptor gamma coactivator 1-alpha* (*PPARGC1A*) (rs8192678) genes have previously been associated with elite athletic performance. This study assessed the influence of polymorphisms in these candidate genes towards endurance test performance in 46 players from a single Australian Football League (AFL) team. Each player provided saliva buccal swab samples for DNA analysis and genotyping and were required to perform two independent two-kilometre running time-trials, six weeks apart. Linear mixed models were created to account for repeated measures over time and to determine whether player genotypes are associated with overall performance in the two-kilometre time-trial. The results showed that the *ADRB1 Arg389Gly* CC (*p* = 0.034) and *PPARGC1A Gly482Ser* GG (*p* = 0.031) genotypes were significantly associated with a faster two-kilometre time-trial. This is the first study to link genetic polymorphism to an assessment of endurance performance in Australian Football and provides justification for further exploratory or confirmatory studies.

## 1. Introduction

Australian Football (AF) is a multi-dimensional team sport, which requires a combination of endurance, strength, power, speed, and competency in sport-specific skills including kicking, handballing, marking, and tackling [1,2,3,4,5,6,7]. The Australia Football League (AFL) represents an elite AF competition and has playing times of four 20-min quarters with time on, with games often spanning beyond 120 min due to stoppages (or, during COVID-19-modified seasons, four 16-min quarters with time on, often spanning 100 min due to stoppages). With an oval playing field of 135 to 185 m in length and 110 to 155 m in width across the competition [8], players consistently run more than 13 km during a typical game [9,10]. Accordingly, AF is characterised as an endurance sport consisting of multiple high-intensity and moderate-intensity efforts [11,12,13,14,15,16,17], with no movement restrictions on footballers during active play. However, positional differences in movement and match-play profiles exist, with players commonly grouped as nomadic (i.e., high running volumes covering the entire playing surface, such as midfielders) or non-nomadic (key positions, such as ruckmen, forwards, and backs) players [14]. Due to these unique qualities, there are many athletic abilities required to be successful within the elite AFL competition.

Athletic ability and performance can be influenced by multiple variables, including environmental factors such as training history, nutrition, body morphology, cognitive factors, and injury susceptibility. Recently, the genetic underpinning of athletic performance in elite athletes is gaining ascendency to understand the predictability of an athlete’s capacity to perform under various constraints in addition to the potential trainability or responsiveness of athletes to various strength and conditioning modalities. Genetics can affect strength, power, and endurance, additional to other traits such as muscle fibre size and composition, flexibility, and neuromuscular coordination [18,19,20,21,22,23,24,25]. Athletic status is at least a partially inheritable trait, with upwards of 66% of athlete variance being explained via genetics [26]. Interest in how an individual’s genotype can impact phenotypes related to athletic performance has gained traction in recent times in AF. Some of the first identified and most influential candidate genes associated with athletic performance include *angiotensin-converting enzyme* (*ACE*), *alpha-actinin-3* (*ACTN3*), *adrenoceptor-beta-1* (*ADRB1*), and *peroxisome proliferator-activated receptor gamma coactivator 1-alpha* (*PPARGC1A*) [27].

Variants within the *ACE* and *ACTN3* genes have been associated with endurance, strength, and power. The *ACE* enzyme regulates fluid volume within the *renin–angiotensin–aldosterone* system (*RAS*) [28,29]. Within intron 16 of the *ACE* gene is an insertion/deletion (I/D) polymorphism (rs4343) of an Alu repetitive element, with the I allele being associated with a lower level of *ACE* enzyme activity [30]. An abundance of evidence indicates that the insertion polymorphism and I allele are associated with elite endurance status in single disciplinary sports such as running (ranging from middle distance to ultramarathon) [31,32], triathletes [33], and rowers [34,35]. The DD genotype has also been found to be beneficial to sprinters [36]. The *ACTN3* protein contributes to the formation of skeletal muscle fibres [37], and helps coordinate type II fast-twitch muscle fibre contraction [22,23,37]. The *R577X* polymorphism (rs1815739) within the *ACTN3* gene can encode for a premature stop codon (T allele), which is associated with improved endurance performance [22,38]. The TT genotype frequencies were found to be significantly lower in bodybuilders and power athletes [39], whereas the T allele and TT genotype frequencies are likely higher in endurance athletes such as endurance running [40,41,42], road cyclists [40], and rowers [40]. Research into the influence of the *ACTN3 R577X* polymorphism in sports has found higher frequencies of the C allele and CC genotype in strength, power, or speed sports such as speed skating [43], track sprinters [36,41,42,44], and field athletes [41,42]. The *ACTN3* gene has also been investigated in soccer, with Santiago et al. [45] finding that the CC and CT genotypes were significantly higher. 

Variants within the *ADRB1 Arg389Gly* (rs1801253) and *PPARGC1A Gly482Ser* (rs8192678) genes have also been linked with endurance performance [24,25,46,47,48,49,50,51,52]. The *ADRB1* gene encodes for G-coupled receptors in cardiac tissue that impacts cardiac output [25,53]. Due to this, most research into *ADRB1* genetic variation has been conducted in patients with cardiac conditions, such as idiopathic or ischemic cardiomyopathy. For example, Wagoner, Craft, Zengel, McGuire, Rathz, Dorn, and Liggett [52] investigated a variant (rs1801253) within the *ADRB1* gene and found that patients with the C allele demonstrated an increase in maximum rate of oxygen consumption (VO2max), exercise time, and endurance performance. However, Wessner et al. [54] found that the GG genotype of this *ADRB1* variant was more prevalent in international or highest national level handball and soccer players. The *PPARGC1A* gene is involved in glucose regulation and lipid metabolism, as well as determination of fibre type and skeletal muscle fibre formation [24,46]. The *PPARGC1A Gly482Ser* variant within this gene has been associated with athletic performance, with the A allele found to be at a lower frequency in Israeli endurance runners, with significant differences between those endurance runners and sprinters of the same level [55]. In addition, the AA genotype was found to be the more favourable genotype for a population of Russian and Lithuanian powerlifters compared to controls [56]. In a meta-analysis of the *PPARGC1a* rs8192678 variant [57], the A allele and the AA genotype were suggested to be beneficial for athletic performance regardless of the type of sport; however, studies in AF are lacking. 

Due to the potential influence of these candidate genetic variants on endurance performance in athletic pursuits and non-elite AF populations, the primary purpose of this study was to investigate any associations of the *ACE* (rs4343), *ACTN3* (rs1815739), *ADRB1* (rs1801253), and *PPARGC1A* (rs8192678) polymorphisms with two-kilometre time-trial endurance test performance in elite AF players. The secondary purpose of the study was to determine if there was a genetic difference between nomadic and non-nomadic players, to examine if particular genotypes were more favourable for certain positions.

## 2. Materials and Methods

### 2.1. Participants

Forty-six (*n* = 46) elite male AF players recruited from an AFL football club participated in the study. All players were injury-free at the time of testing. To ensure anonymity, players were assigned a randomised, non-identifiable code. All players were provided with information letters outlining the purpose of the study, along with its potential benefits and risks, and provided written informed consent for their participation. The study was approved by the Edith Cowan University Human Research and Ethics Committee (ID: 2019-00181-JACOB).

### 2.2. Sample Collection and DNA Analysis

Buccal saliva samples were collected via mouth swabs with participants instructed to brush the edge of a soft tip swab along the insides of their cheek and gums for 30 s [58,59]. Players were asked not to consume coffee, alcohol, or food two hours prior to saliva collection. Collected samples were labelled with a numeric code for de-identification and were sent to the Australian Genome Research Facility (AGRF; Brisbane, QLD, Australia; NATA 17025) for DNA extraction and genotyping using the Agena Bioscience MassARRAY system (AGRF). A summary of the genetic variants investigated in this study is presented in Table 1.

### 2.3. Endurance Testing

Endurance performance was evaluated twice to obtain accurate and reliable results for two-kilometre time-trials performed six weeks apart. Trials were conducted on a certified athletics track (Oceania Athletics Association) during pre-season training in conjunction with the football club’s regular pre-season testing. During the six-week training block, players participated in the same structured pre-season training program overseen by the club’s high-performance department. All times were visually verified through video recording in conjunction with the AFL club’s guidelines for two-kilometre time-trials.

### 2.4. Statistical Analysis

Data were statistically analysed using SPSS V.24 (IBM, Armonk, NY, USA). A generalised linear mixed model (GLMM) was created to analyse the relationship between covariates and overall performance in the two-kilometre time-trial. Separate GLMMs were created to account for repeated measures over time, and to determine whether player genotypes are associated with overall performance in the two-kilometre time-trial. Beta (β) coefficients have been reported as a standardised measure of effect (i.e., effect size) for the GLMMs. Further analysis was completed with the genotype and allele frequencies, which were compared using Pearson’s Chi-square (χ2) tests between nomadic and non-nomadic positions, as well as T-tests, or a non-parametric alternative, to determine the mean difference between groups. A significant nominal *p*-value of <0.05 was employed. ETA square values (η^2^) for Chi-square analyses were also calculated to determine the effect of any observed associations, defined as none (η^2^ < 0.010), small (η^2^ < 0.060), moderate (η^2^ < 0.140), and large (η^2^ < 0.200).

## 3. Results

This study is preliminary in nature with access to a single AFL football team producing a sample size of 46 elite AF players. Demographic characteristics of these male players were: age = 24.4 ± 4.0 years; weight = 88.3 ± 8.1 kg; height = 187.8 ± 6.3 cm; body mass index (BMI) = 24.9 ± 1.4. Two-kilometre time-trials produced completion times of 406.9 (± 22.0) seconds for the first trial and 400.9 (± 17.0) seconds for the second trial. The players’ genotype and allele frequencies are presented in Table 1.

Age (*p* = 0.750; 95% CI [−1.191, 0.861]), height (*p* = 0.086; 95% CI [−6.964–104.060]), weight (*p* = 0.110; 95% CI [−0.086–0.827), and BMI (*p* = 0.674; 95% CI [−2.164, 3.332]) were not significantly associated with two-kilometre performance (Table 2). Therefore, these variables were not considered as covariates in subsequent GLMMs investigating individual genetic variants.

To account for repeated measures, separate GLMMs between the first time-trial (time point 1) and the second time-trial (time point 2) revealed that *ADRB1 Arg389Gly* and *PPARGC1a Gly482Ser* variants were significantly associated with two-kilometre time-trial performance (Table 3). Participants with the *ADRB1 Arg389Gly* CC genotype were 17.568 s faster (*p* = 0.034; 95% CI [−33.766, −1.371]) in the two-kilometre time-trial when compared to participants carrying the GG genotype. Similarly, individuals with the *PPARGC1a Gly482Ser* GG genotype were 14.421 s (*p* = 0.031; 95% CI [1.311, 27.531]) faster in the two-kilometre time-trial than individuals with the AA genotype.

Nomadic players had an average height of 185.08 ± 4.58 cm, average weight of 84.78 ± 5.61 kg, and average BMI of 24.75 ± 1.37. Non-nomadic players had an average height of 197.35 ± 3.36 cm, average weight of 100.57 ± 5.36 kg, and average BMI of 25.83 ± 1.27. A T-test was conducted been nomadic and non-nomadic players for height (*p* = 0.312; 95% CI [−15.234–−9.312]), while Mann–Whitney tests were conducted for weight and BMI. Non-nomadic players scored higher in weight (Mdn = 40.58) and BMI (Mdn = 30.54) than nomadic players (weight: Mdn = 18.31; BMI: Mdn = 21.76; weight: U = 409.00; *p* = 0.00; BMI: U = 288.500; *p* = 0.048). There was a significant difference in *ADRB1 Arg389Gly* genotype frequency between nomadic and non-nomadic positions (χ^2^ = 6.293, *p* = 0.037), with the CC genotype being significantly overrepresented in nomadic positions (63.6%) compared with non-nomadic positions (25.0%; Table 4). Furthermore, the C allele was significantly overrepresented in nomadic positions (80.3%) compared to non-nomadic positions (19.7%; χ^2^ = 6.148, *p* = 0.017).

## 4. Discussion

This preliminary study investigates the frequency of genotypes from a group of candidate genes, which may contribute to the differences in endurance performance of elite AF players. The study further investigated the presence of any associations between candidate variants and performance in the two-kilometre time-trial for elite AF players. This study is the first to investigate the frequencies of the *ADRB1 Arg389Gly* and *PPARGC1a Gly482Ser* variants in elite AF players. The C allele of *ADRB1 Arg389Gly* and the G allele of *PPARGC1a Gly482Ser* had higher frequencies than their respective allele counterparts, with the homozygous genotypes for those alleles also having a greater frequency than the other genotypes. Secondly, the results from the current study found a significant association between two-kilometre time-trial performance and *ADRB1 Arg389Gly* and *PPARGC1a Gly482Ser* variants, indicating these may contribute to endurance performance. 

The *ADRB1* gene encodes for the beta-adrenergic receptor, with stimulation resulting in the activation and phosphorylation of targeted proteins in cardiac tissue, regulating cardiac function [60,61,62]. In previous literature, positive associations between the C allele of the *ADRB1 Arg389Gly* polymorphism have been seen in aerobic capacity performance in heart disease populations [52]. Sawczuk et al. [63] did find an association of the haplotype including loci *Arg389Gly* and *Ser49Gly*, showing that the *49Gly:Arg389* carriers had a positive association with endurance performance. In a sporting context, a group of Austrian handball and soccer players were found to have a higher frequency of the *Arg389Gly* GG genotype; however, its effect on endurance performance was not investigated further [54]. Despite differences between the current study and previous literature, the association between the C allele of *ADRB1 Arg389Gly* and the improvement in testing outcome, along with the higher frequency of the *Arg389Gly* CC genotype and *Arg389Gly* C allele in the elite AF population provides evidence that the *Arg389Gly* C allele may be the preferred allele in AF. Nonetheless, with the conflicting research around *ADRB1*, further investigation is required. 

The *PPARCG1a Gly482Ser* variant was also significantly associated with two-kilometre time-trial performance in our study. When accounting for repeated measures, individuals with the AA genotype of the *Gly482Ser* variant were predicted to perform 14.46 s slower on the time-trial. These results are supported by a study with Turkish elite endurance athletes, where participants with the A allele were found to have poorer endurance performance and aerobic capacity [64]. Similar results were found in Maciejewska, Sawczuk, Cieszczyk, Mozhayskaya, and Ahmetov [48] who found that the G allele of the *PPARGC1a Gly482Ser* polymorphism was associated with elite endurance performance in a Polish population. However, studies conducted in Chinese populations have found no association [65] between endurance performance and the *PPARGC1a Gly482Ser* polymorphism; however, this could be explained by the different frequencies of the variant in Chinese populations. Further studies found that the “less optimal” genotype of AA had better performance times in a cohort of elite endurance triathletes [66,67]. As AF players were of primarily European descent, the results from the study still indicate that ethnicity may impact the phenotypic response of the *PPARGC1a* polymorphism.

The current study showed that there was no significant association of *ACE I/D* variant and performance in time-trial results. The II genotype is associated with lower *ACE* plasma levels [68], leading to greater dilation through the vascular system, thus increasing cardiac output [69,70,71,72]. However, in sub-elite AF players, the deletion polymorphism was found to positively affect endurance performance, as well as the more commonly expected 20-metre sprint and vertical performance [59]. Heffernan et al. [73] found no difference in the *ACE I/D* polymorphism between rugby union players and Caucasian controls, while Magi et al. [74] found that the *ACE I/D* gene was more prevalent in young cross-country skiers, with lower frequencies of the DD genotype. 

We previously reported that the frequency of the T allele of the *ACTN3 R577X* variant is underreported in elite AF [75,76], reflecting the results of Massidda, Bachis, Corrias, Piras, Scorcu, Culigioni, Masala, and Calo [76] who found no significance in *ACTN3* polymorphism distribution in Italian team sports athletes, endurance athletes, and healthy controls. However, Santiago, Gonzalez-Freire, Serratosa, Morate, Meyer, Gomez-Gallego, and Lucia [45] found CC and CT genotypes to be significantly higher in soccer players, indicating that the C allele may be more beneficial to soccer players and potentially other team sports. However, when examining the effect of this genotype on endurance capacity, it was not significantly associated with two-kilometre time-trial performance. This finding is in part supported by Silva et al. [77] who found those with the TT genotype had a greater baseline VO2max; however, after undergoing their training protocol, no difference between TT and CC genotypes was illustrated while investigating the effects of endurance training on healthy adult males. This may indicate a limit to where carriage of the TT genotype is of benefit once similar training protocols are partaken. However, over a five-year period in junior Estonian cross-country skiers, an increase in maximal oxygen uptake peak (VO2peak) was seen for males with the TT genotype [74]. In contrast, in elite Brazilian soccer players, the players with the TT genotype had significantly higher estimated maximal oxygen consumption (VO2max) using the Yo-Yo intermittent endurance test than their CC counterparts [78]. Even though the results from our study regarding *ACTN3* need further investigation, the significantly higher frequency of the C allele provides initial evidence that it may be more beneficial for an AF player to have either the CC or CT genotype to become elite.

Another finding from the current study was the significant difference between the nomadic and non-nomadic positions and the *ADRB1 Arg389Gly* genotypes. To our knowledge, this is the first study to investigate the genetic differences in AF players playing in different positions. Playing positions were defined and grouped by the AFL club, with nomadic players being those who followed the play across the full oval. This supports the previous findings of this study, suggesting that the *Arg389Gly* CC genotype and the *Arg389Gly* C allele are a potential benefit to endurance performance and furthermore, to elite AF players in general. Due to the nature of nomadic positions, there can be an assumption that players who fall into this category will have greater endurance performance capacities because of the demands of the position. However, this does not account for the possible genetic impact of the *ADRB1 Arg389Gly* C allele.

## 5. Limitations of the Study

Complete access to an elite AFL squad was provided, and is a strength of the study. However due to the elite nature of the cohort and the novel, exploratory nature of the study, the sample size of the participants is relatively low and these findings in Australian Football may require replication with larger sample sizes across multiple teams or the entire AFL competition. Furthermore, to account for individual variability in performance of the two-kilometre time-trial, multiple measures were taken to ensure consistent and reliable performance necessary for genetic association. While more precise measures of VO2max using a laboratory setting could have been used to test endurance performance, the validity of the two-kilometre time-trial should be noted with its routine use in elite AF, including the AFL National Draft Combine, which is conducted every season prior to the draft. Though the investigators are confident in the training partaken and the synchronicity of it, the inability to dictate a precise training schedule and method across all players is a limitation. This study was limited to investigating genetic associations with endurance performance. Each genetic variant was investigated singularly; however, it is known that often, a combination of genes and genotypes provides the phenotypic outcome.

## 6. Conclusions

The C allele of the *ADRB1 Arg389Gly* variant and the homozygous GG genotype of the *PPARGC1a Gly482Ser* variant were significantly associated with two-kilometre time-trial performance in AFL players. The CC *ADRB1 Arg389Gly* genotype was found to have a better improvement in repeated two-kilometre time-trials compared to the GG genotype, while the GG *PPARGC1a Gly482Ser* genotype had a poorer performance. These results may indicate a preferred genotype for endurance running performance in elite Australian Footballers. Additional analysis also discovered the *ADRB1 Arg389Gly* C allele was significantly different for characterised nomadic player positions relative to non-nomadic key positions. Further research into possible interactions and combinations of multiple variants and their influence on endurance performance outcomes, and differences between precise playing positions appear to be a logical next step in this research field. Future research into the genetic distribution of elite AF players and the genotype and allelic impact on endurance performance should utilise controlled trials with specifically prescribed training measures to provide high-quality insights. Further studies should also look to increase the sample size of elite cohorts for characterisation by engaging multiple teams in the AFL competition; through controlled trials, this may improve practitioner understanding of the athletic potential of individual footballers. Insights into the contribution of genetic variants and training response to various fitness parameters are a high priority for sport scientists, which may enable the preparation of high fidelity and highly targeted strength and conditioning programs with precision exercise prescription based on a specific footballer’s trainability and training response relative to genetic characteristics—a long-term aspiration of this line of research.

## Figures and Tables

**Table 1:** Variant distribution in elite Australian Football (AF) athletes.

		Elite AF *n* (%)
*ACTN3 R577X*	CC	21 (45.7%)
	CT	23 (50.0%)
	TT	2 (4.3%)
	C allele	65 (70.7%)
	T allele	27 (29.3%)
*ACE I/D*	II	11 (23.9%)
	ID	23 (50.0%)
	DD	12 (26.1%)
	I allele	45 (48.9%)
	D allele	47 (51.1%)
*ADRB1 Arg389Gly*	CC	25 (54.3%)
	CG	18 (39.1%)
	GG	3 (6.5%)
	C allele	68 (73.9%)
	G allele	24 (26.1%)
*PPARGC1a Gly482Ser*	GG	23 (50.0%)
	GA	18 (39.1%)
	AA	5 (10.9%)
	G allele	64 (69.6%)
	A allele	28 (30.4%)

**Table 2:** Generalised linear mixed model of covariates and the two-kilometre time-trial time.

Variable	β Coefficient	Standard Error	t Value	Significance	95% CI
Time	5.467	4.184	1.307	0.195	−2.847–13.781
Age	−0.165	0.516	−0.320	0.750	−1.191–0.861
Height	0.524	0.302	1.735	0.086	−0.076–1.124
Weight	0.378	0.231	1.635	0.106	−0.082–0.838
BMI	0.584	1.382	0.423	0.674	−2.164–3.332

Note: β coefficient = standardised effect size.

**Table 3:** Generalised linear model of genetic variables and the two-kilometre time-trial time.

Variable		β Coefficient	Standard Error	Significance	95% CI
***ADRB1***					
Intercept		416.292	7.826	0.000	400.738–431.849
Time point	1	5.467	4.128	0.189	−2.740–13.673
	2	0 *			
*ADRB1 Arg389Gly*	CC	−17.568	8.128	**0.034**	−33.766–−1.371
	CG	−13.806	8.297	0.100	−30.301–2.688
	GG	0*			
***ACE***					
Intercept		397.802	4.403	0.000	389.049–406.556
Time point	1	5.467	4.198	0.196	−2.878–13.812
	2	0 *			
*ACE I/D*	II	6.049	5.815	0.301	−5.510–17.608
	ID	4.146	4.999	0.409	−5.791–14.084
	DD	0 *			
***ACTN3***					
Intercept		399.571	9.807	0.00	380.074–419.067
Time point	1	5.467	4.222	0.199	−2.927–13.860
	2	0 *			
*ACTN3 R577X*	CC	0.748	10.153	0.941	−19.436–20.932
	CT	2.929	10.093	0.772	−17/135–22.993
	TT	0 *			
***PPARGC1a***					
Intercept		389.763	6.175	0.00	377.488–402.038
Time point	1	5.467	4.111	0.187	−2.706–13.640
	2	0 *			
*PPARGC1a Gly482Ser*	GG	14.421	6.595	**0.031**	1.311–27.531
	AG	11.293	6.800	0.100	−2.224–24.810
	AA	0*			

Note: Significant effects are bolded. * indicated comparison group. β coefficient = standardised effect size.

**Table 4:** Genotype and allele distribution between nomadic and non-nomadic positional categories.

		Nomadic *n* (%)	Non-Nomadic *n* (%)	Significance (*p*)	ETA Squared (η^2^)	
*ACE I/D*	II	8 (24.2%)	3 (25.0%)			
	ID	16 (48.5%)	7 (58.3%)	0.904	0.013	Small
	DD	9 (27.3%)	2 (16.7%)			
	I allele	32 (48.5%)	13 (54.2%)	0.812	0.005	Small
	D allele	34 (51.5%)	11 (45.8%)			
*ACTN3 R577X*	CC	14 (42.4%)	6 (50.0%)			
	CT	17 (51.5%)	6 (50.0%)	1.000	0.019	Small
	TT	2 (6.1%)	0 (0.0%)			
	C allele	45 (68.2%)	18 (75.0%)	0.611	0.009	None
	T allele	21 (31.8%)	6 (25.0%)			
*ADRB1*	CC	21 (63.6%)	3 (25.0%)			
*Arg389Gly*	CG	11 (33.3%)	7 (58.3%)	**0.037**	0.140	Large
	GG	1 (3.0%)	2 (16.7%)			
	C allele	53 (80.3%)	13 (54.2%)	**0.029**	0.137	Moderate
	G allele	13 (19.7%)	11 (45.8%)			
*PPARGC1a*	GG	17 (51.5%)	6 (50.0%)			
*Gly482Ser*	AG	11 (33.3%)	6 (50.0%)	0.393	0.055	Small
	AA	5 (15.2%)	0 (0.0%)			
	G allele	45 (68.2%)	18 (75.0%)	0.611	0.009	None
	A allele	21 (31.8%)	6 (25.0%)			

Note: Significant effects are bolded.

## Data Availability

The data presented in this study are available on request from the corresponding authors. The data are not publicly available.

## References

[1] Robertson S., Woods C., Gastin P. (2015). Predicting higher selection in elite junior Australian Rules football: The influence of physical performance and anthropometric attributes. J. Sci. Med. Sport.

[2] Woods C.T., Raynor A.J., Bruce L., McDonald Z., Collier N. (2015). Predicting playing status in junior Australian Football using physical and anthropometric parameters. J. Sci. Med. Sport.

[3] Hart N.H., Nimphius S., Cochrane J.L., Newton R.U. (2013). Leg mass characteristics of accurate and inaccurate kickers: An Australian football perspective. J. Sports Sci..

[4] Hart N.H., Nimphius S., Spiteri T., Cochrane J.L., Newton R.U. (2016). Relationship between Leg Mass, Leg Composition and Foot Velocity on Kicking Accuracy in Australian Football. J. Sports Sci. Med..

[5] Hart N.H., Nimphius S., Spiteri T., Newton R.U. (2014). Leg strength and lean mass symmetry influences kicking performance in Australian football. J. Sports Sci. Med..

[6] Hart N.H., Nimphius S., Weber J., Spiteri T., Rantalainen T., Dobbin M., Newton R.U. (2016). Musculoskeletal Asymmetry in Football Athletes: A Product of Limb Function over Time. Med. Sci. Sports Exerc..

[7] Hart N.H., Spiteri T., Lockie R.G., Nimphius S., Newton R.U. (2014). Detecting deficits in change of direction performance using the preplanned multidirectional Australian football league agility test. J. Strength Cond. Res..

[8] AFL (2016). Laws of Australian Football.

[9] Colby M.J., Dawson B., Heasman J., Rogalski B., Rosenberg M., Lester L., Peeling P. (2017). Preseason Workload Volume and High-Risk Periods for Noncontact Injury Across Multiple Australian Football League Seasons. J. Strength Cond. Res..

[10] Kelly S.J., Watsford M.L., Rennie M.J., Spurrs R.W., Austin D., Pine M.J. (2019). Match-play movement and metabolic power demands of elite youth, sub-elite and elite senior Australian footballers. PLoS ONE.

[11] Boyd L.J., Ball K., Aughey R.J. (2013). Quantifying external load in Australian football matches and training using accelerometers. Int. J. Sports Physiol. Perform..

[12] Coutts A.J., Quinn J., Hocking J., Castagna C., Rampinini E. (2010). Match running performance in elite Australian Rules Football. J. Sci. Med. Sport.

[13] Dawson B., Hopkinson R., Appleby B., Stewart G., Roberts C. (2004). Player movement patterns and game activities in the Australian Football League. J. Sci. Med. Sport.

[14] Gray A.J., Jenkins D.G. (2010). Match analysis and the physiological demands of Australian football. Sports Med..

[15] Haycraft J.A.Z., Kovalchik S., Pyne D.B., Robertson S. (2017). Physical characteristics of players within the Australian Football League participation pathways: A systematic review. Sports Med. Open.

[16] Veale J.P., Pearce A.J., Carlson J.S. (2007). Player movement patterns in an elite junior Australian rules football team: An exploratory study. J. Sports Sci. Med..

[17] Wisbey B., Montgomery P.G., Pyne D.B., Rattray B. (2010). Quantifying movement demands of AFL football using GPS tracking. J. Sci. Med. Sport.

[18] Drozdovska S.B., Dosenko V.E., Ahmetov I.I., Ilyin V.N. (2013). The Association of Gene Polymorphisms with Athlete Status in Ukrainians. Biol. Sport.

[19] Gineviciene V., Jakaitiene A., Tubelis L., Kucinskas V. (2014). Variation in the ACE, PPARGC1A and PPARA genes in Lithuanian football players. Eur. J. Sport Sci..

[20] Mustafina L.J., Naumov V.A., Cieszczyk P., Popov D.V., Lyubaeva E.V., Kostryukova E.S., Fedotovskaya O.N., Druzhevskaya A.M., Astratenkova I.V., Glotov A.S. (2014). AGTR2 gene polymorphism is associated with muscle fibre composition, athletic status and aerobic performance. Exp. Physiol..

[21] Puthucheary Z., Skipworth J.R., Rawal J., Loosemore M., Van Someren K., Montgomery H.E. (2011). The ACE gene and human performance: 12 years on. Sports Med..

[22] Pickering C., Kiely J. (2017). ACTN3: More than Just a Gene for Speed. Front. Physiol..

[23] Yang N., MacArthur D.G., Gulbin J.P., Hahn A.G., Beggs A.H., Easteal S., North K. (2003). ACTN3 genotype is associated with human elite athletic performance. Am. J. Hum. Genet..

[24] Lin J., Wu H., Tarr P.T., Zhang C.Y., Wu Z., Boss O., Michael L.F., Puigserver P., Isotani E., Olson E.N. (2002). Transcriptional co-activator PGC-1 alpha drives the formation of slow-twitch muscle fibres. Nature.

[25] Taylor M.R. (2007). Pharmacogenetics of the human beta-adrenergic receptors. Pharm. J..

[26] De Moor M.H., Spector T.D., Cherkas L.F., Falchi M., Hottenga J.J., Boomsma D.I., De Geus E.J. (2007). Genome-wide linkage scan for athlete status in 700 British female DZ twin pairs. Twin Res. Hum. Genet..

[27] Jacob Y., Spiteri T., Hart N.H., Anderton R.S. (2018). The Potential Role of Genetic Markers in Talent Identification and Athlete Assessment in Elite Sport. Sports.

[28] Bernstein K.E., Ong F.S., Blackwell W.L., Shah K.H., Giani J.F., Gonzalez-Villalobos R.A., Shen X.Z., Fuchs S., Touyz R.M. (2013). A modern understanding of the traditional and nontraditional biological functions of angiotensin-converting enzyme. Pharmacol. Rev..

[29] Zhang Z., Xu G., Liu D., Fan X., Zhu W., Liu X. (2012). Angiotensin-converting enzyme insertion/deletion polymorphism contributes to ischemic stroke risk: A meta-analysis of 50 case-control studies. PLoS ONE.

[30] Woods D.R., Pollard A.J., Collier D.J., Jamshidi Y., Vassiliou V., Hawe E., Humphries S.E., Montgomery H.E. (2002). Insertion/deletion polymorphism of the angiotensin I-converting enzyme gene and arterial oxygen saturation at high altitude. Am. J. Respir. Crit. Care Med..

[31] Myerson S., Hemingway H., Budget R., Martin J., Humphries S., Montgomery H. (1999). Human angiotensin I-converting enzyme gene and endurance performance. J. Appl. Physiol..

[32] Nazarov I.B., Woods D.R., Montgomery H.E., Shneider O.V., Kazakov V.I., Tomilin N.V., Rogozkin V.A. (2001). The angiotensin converting enzyme I/D polymorphism in Russian athletes. Eur. J. Hum. Genet..

[33] Shenoy S., Tandon S., Sandhu J., Bhanwer A.S. (2010). Association of Angiotensin Converting Enzyme gene Polymorphism and Indian Army Triathletes Performance. Asian J. Sports Med..

[34] Cieszczyk P., Krupecki K., Maciejewska A., Sawczuk M. (2009). The angiotensin converting enzyme gene I/D polymorphism in Polish rowers. Int. J. Sports Med..

[35] Gayagay G., Yu B., Hambly B., Boston T., Hahn A., Celermajer D.S., Trent R.J. (1998). Elite endurance athletes and the ACE I allele--the role of genes in athletic performance. Hum. Genet..

[36] Papadimitriou I.D., Lucia A., Pitsiladis Y.P., Pushkarev V.P., Dyatlov D.A., Orekhov E.F., Artioli G.G., Guilherme J.P., Lancha A.H., Gineviciene V. (2016). ACTN3 R577X and ACE I/D gene variants influence performance in elite sprinters: A multi-cohort study. BMC Genom..

[37] Broos S., Malisoux L., Theisen D., Francaux M., Deldicque L., Thomis M.A. (2012). Role of alpha-actinin-3 in contractile properties of human single muscle fibers: A case series study in paraplegics. PLoS ONE.

[38] Eynon N., Hanson E.D., Lucia A., Houweling P.J., Garton F., North K.N., Bishop D.J. (2013). Genes for elite power and sprint performance: ACTN3 leads the way. Sports Med..

[39] Roth S.M., Walsh S., Liu D., Metter E.J., Ferrucci L., Hurley B.F. (2008). The ACTN3 R577X nonsense allele is under-represented in elite-level strength athletes. Eur. J. Hum. Genet..

[40] Eynon N., Ruiz J.R., Femia P., Pushkarev V.P., Cieszczyk P., Maciejewska-Karlowska A., Sawczuk M., Dyatlov D.A., Lekontsev E.V., Kulikov L.M. (2012). The ACTN3 R577X polymorphism across three groups of elite male European athletes. PLoS ONE.

[41] Niemi A.K., Majamaa K. (2005). Mitochondrial DNA and ACTN3 genotypes in Finnish elite endurance and sprint athletes. Eur. J. Hum. Genet..

[42] Papadimitriou I.D., Papadopoulos C., Kouvatsi A., Triantaphyllidis C. (2008). The ACTN3 gene in elite Greek track and field athletes. Int. J. Sports Med..

[43] Ahmetov I.I., Druzhevskaya A.M., Lyubaeva E.V., Popov D.V., Vinogradova O.L., Williams A.G. (2011). The dependence of preferred competitive racing distance on muscle fibre type composition and ACTN3 genotype in speed skaters. Exp. Physiol..

[44] Eynon N., Alves A.J., Yamin C., Sagiv M., Duarte J.A., Oliveira J., Ayalon M., Goldhammer E., Sagiv M., Meckel Y. (2009). Is there an ACE ID-ACTN3 R577X polymorphisms interaction that influences sprint performance?. Int. J. Sports Med..

[45] Santiago C., Gonzalez-Freire M., Serratosa L., Morate F.J., Meyer T., Gomez-Gallego F., Lucia A. (2008). ACTN3 genotype in professional soccer players. Br. J. Sports Med..

[46] Charos A.E., Reed B.D., Raha D., Szekely A.M., Weissman S.M., Snyder M. (2012). A highly integrated and complex PPARGC1A transcription factor binding network in HepG2 cells. Genome Res..

[47] Eynon N., Duarte J.A., Oliveira J., Sagiv M., Yamin C., Meckel Y., Sagiv M., Goldhammer E. (2009). ACTN3 R577X polymorphism and Israeli top-level athletes. Int. J. Sports Med..

[48] Maciejewska A., Sawczuk M., Cieszczyk P., Mozhayskaya I.A., Ahmetov I.I. (2012). The PPARGC1A gene Gly482Ser in Polish and Russian athletes. J. Sports Sci..

[49] Moore G.E., Shuldiner A.R., Zmuda J.M., Ferrell R.E., McCole S.D., Hagberg J.M. (2001). Obesity gene variant and elite endurance performance. Metabolism.

[50] Rubio J.C., Martin M.A., Rabadan M., Gomez-Gallego F., San Juan A.F., Alonso J.M., Chicharro J.L., Perez M., Arenas J., Lucia A. (2005). Frequency of the C34T mutation of the AMPD1 gene in world-class endurance athletes: Does this mutation impair performance?. J. Appl. Physiol..

[51] Santiago C., Ruiz J.R., Buxens A., Artieda M., Arteta D., Gonzalez-Freire M., Rodriguez-Romo G., Altmae S., Lao J.I., Gomez-Gallego F. (2011). Trp64Arg polymorphism in ADRB3 gene is associated with elite endurance performance. Br. J. Sports Med..

[52] Wagoner L.E., Craft L.L., Zengel P., McGuire N., Rathz D.A., Dorn G.W., Liggett S.B. (2002). Polymorphisms of the beta1-adrenergic receptor predict exercise capacity in heart failure. Am. Heart J..

[53] Johnson J.A., Liggett S.B. (2011). Cardiovascular pharmacogenomics of adrenergic receptor signaling: Clinical implications and future directions. Clin. Pharmacol. Therepeutics.

[54] Wessner B., Stuparits P., Fail C., Pavic F., Tschan H., Bachl N. (2016). Genetic polymorphisms in alpha-actinin 3 and adrenoceptor beta genes in Austrian elite athletes and healthy controls. Swiss Sports Exerc. Med..

[55] Eynon N., Meckel Y., Sagiv M., Yamin C., Amir R., Sagiv M., Goldhammer E., Duarte J.A., Oliveira J. (2009). Do PPARGC1A and PPARalpha polymorphisms influence sprint or endurance phenotypes?. Scand. J. Med. Sci. Sports.

[56] Gineviciene V., Jakaitiene A., Aksenov M.O., Aksenova A.V., Druzhevskaya A.M., Astratenkova I.V., Egorova E.S., Gabdrakhmanova L.J., Tubelis L., Kucinskas V. (2016). Association analysis of ACE, ACTN3 and PPARGC1A gene polymorphisms in two cohorts of European strength and power athletes. Biol. Sport.

[57] Chen Y., Wang D., Yan P., Yan S., Chang Q., Cheng Z. (2019). Meta-analyses of the association between the PPARGC1A Gly482Ser polymorphism and athletic performance. Biol. Sport.

[58] Jacob Y., Chivers P., Anderton R.S. (2019). Genetic predictors of match performance in sub-elite Australian football players: A pilot study. J. Exerc. Sci. Fit..

[59] Jacob Y., Cripps A., Evans T., Chivers P.T., Joyce C., Anderton R.S. (2016). Identification of genetic markers for skill and athleticism in sub-elite Australian football players: A pilot study. J. Sports Med. Phys. Fit..

[60] Baruscotti M., Barbuti A., Bucchi A. (2010). The cardiac pacemaker current. J. Mol. Cell. Cardiol..

[61] Ciccarelli M., Santulli G., Pascale V., Trimarco B., Iaccarino G. (2013). Adrenergic receptors and metabolism: Role in development of cardiovascular disease. Front. Physiol..

[62] Woo A.Y., Xiao R.P. (2012). beta-Adrenergic receptor subtype signaling in heart: From bench to bedside. Acta Pharm. Sin..

[63] Sawczuk M., Maciejewska-Karlowska A., Cieszczyk P., Zarebska A. (2012). Ser49Gly and Arg389Gly polymorphisms of the ADRB1 gene and endurance performance. Cent. Eur. J. Biol..

[64] Tural E., Kara N., Agaoglu S.A., Elbistan M., Tasmektepligil M.Y., Imamoglu O. (2014). PPAR-alpha and PPARGC1A gene variants have strong effects on aerobic performance of Turkish elite endurance athletes. Mol. Biol. Rep..

[65] Varillas Delgado D., Tellería Orriols J.J., Monge Martín D., Del Coso J. (2020). Genotype scores in energy and iron-metabolising genes are higher in elite endurance athletes than in non-athlete controls. Appl. Physiol. Nutr. Metab..

[66] Grealy R., Herruer J., Smith C.L., Hiller D., Haseler L.J., Griffiths L.R. (2015). Evaluation of a 7-gene genetic profile for athletic endurance phenotype in ironman championship triathletes. PLoS ONE.

[67] He Z., Hu Y., Feng L., Bao D., Wang L., Li Y., Wang J., Liu G., Xi Y., Wen L. (2008). Is there an association between PPARGC1A genotypes and endurance capacity in Chinese men?. Scand. J. Med. Sci. Sports.

[68] Hassanin O.M., Moustafa M., El Masry T.M. (2014). Association of insertion–deletion polymorphism of ACE gene and Alzheimer’s disease in Egyptian patients. Egypt. J. Med. Hum. Genet..

[69] Hagberg J.M., Moore G.E., Ferrell R.E. (2001). Specific genetic markers of endurance performance and VO2max. Exerc. Sport Sci. Rev..

[70] Vaughan D., Huber-Abel F.A., Graber F., Hoppeler H., Fluck M. (2013). The angiotensin converting enzyme insertion/deletion polymorphism alters the response of muscle energy supply lines to exercise. Eur. J. Appl. Physiol..

[71] Zhang B., Tanaka H., Shono N., Miura S., Kiyonaga A., Shindo M., Saku K. (2003). The I allele of the angiotensin-converting enzyme gene is associated with an increased percentage of slow-twitch type I fibers in human skeletal muscle. Clin. Genet..

[72] Dekany M., Harbula I., Berkes I., Gyore I., Falus A., Pucsok J. (2006). The role of insertion allele of angiotensin converting enzyme gene in higher endurance efficiency and some aspects of pathophysiological and drug effects. Curr. Med. Chem..

[73] Heffernan S.M., Kilduff L.P., Erskine R.M., Day S.H., McPhee J.S., McMahon G.E., Stebbings G.K., Neale J.P., Lockey S.J., Ribbans W.J. (2016). Association of ACTN3 R577X but not ACE I/D gene variants with elite rugby union player status and playing position. Physiol. Genom..

[74] Magi A., Unt E., Prans E., Raus L., Eha J., Veraksits A., Kingo K., Koks S. (2016). The Association Analysis between ACE and ACTN3 Genes Polymorphisms and Endurance Capacity in Young Cross-Country Skiers: Longitudinal Study. J. Sports Sci. Med..

[75] Jacob Y., Hart N.H., Cochrane Wilke J., Spiteri T., Laws S.M., Jones A., Rogalski B., Kenna J., Anderton R.S. (2020). ACTN3 (R577X) Genotype is associated with Australian Football League Players. J. Strength Cond. Res..

[76] Massidda M., Bachis V., Corrias L., Piras F., Scorcu M., Culigioni C., Masala D., Calo C.M. (2015). ACTN3 R577X polymorphism is not associated with team sport athletic status in Italians. Sports Med. Open.

[77] Silva M.S., Bolani W., Alves C.R., Biagi D.G., Lemos J.R., da Silva J.L., de Oliveira P.A., Alves G.B., de Oliveira E.M., Negrao C.E. (2015). Elimination of influences of the ACTN3 R577X variant on oxygen uptake by endurance training in healthy individuals. Int. J. Sports Physiol. Perform..

[78] Pimenta E.M., Coelho D.B., Veneroso C.E., Barros Coelho E.J., Cruz I.R., Morandi R.F., De A.P.G., Carvalho M.R., Garcia E.S., De Paz Fernandez J.A. (2013). Effect of ACTN3 gene on strength and endurance in soccer players. J. Strength Cond. Res..

